# The Newly Synthesized Pyrazole Derivative 5-(1-(3 Fluorophenyl)-1*H*-Pyrazol-4-yl)-2*H*-Tetrazole Reduces Blood Pressure of Spontaneously Hypertensive Rats *via* NO/cGMO Pathway

**DOI:** 10.3389/fphys.2018.01073

**Published:** 2018-08-07

**Authors:** Neidiane R. Trindade, Paulo R. Lopes, Lara M. Naves, James O. Fajemiroye, Pedro H. Alves, Nathalia O. Amaral, Luciano M. Lião, Ana C. S. Rebelo, Carlos H. Castro, Valdir A. Braga, Ricardo Menegatti, Gustavo R. Pedrino

**Affiliations:** ^1^Center for Neuroscience and Cardiovascular Research, Department of Physiological Sciences, Federal University of Goiás, Goiânia, Brazil; ^2^Pharmacy Faculty, Federal University of Goiás, Goiânia, Brazil; ^3^Integrative Laboratory of Cardiovascular and Neurological Pathophysiology, Department of Physiological Sciences, Federal University of Goiás, Goiânia, Brazil; ^4^Institute of Chemistry, Federal University of Goiás, Goiânia, Brazil; ^5^Department of Morphology, Federal University of Goiás, Goiânia, Brazil; ^6^Department of Biotechnology, Biotechnology Center, Federal University of Paraiba, João Pessoa, Brazil

**Keywords:** spontaneously hypertensive rats, pyrazole derivative, antihypertensive drugs, vasodilatation, muscarinic receptors

## Abstract

The search for new antihypertensive drugs has grown in recent years because of high rate of morbidity among hypertensive patients and several side effects that are associated with the first-line medications. The current study sought to investigate the antihypertensive effect of a newly synthesized pyrazole derivative known as 5-(1-(3 fluorophenyl)-1H-pyrazol-4-yl)-2H-tetrazole (LQFM-21). Spontaneously hypertensive rats (SHR) were used to evaluate the effect of LQFM-21 on mean arterial pressure (MAP), heart rate (HR), renal vascular conductance (RVC), arterial vascular conductance (AVC), baroreflex sensitivity (BRS) index, and vascular reactivity. Acute intravenous (iv) administration of LQFM-21 (0.05, 0.1, 0.2, and 0.4 mg kg^-1^) reduced MAP and HR, and increased RVC and AVC. Chronic oral administration of LQFM-21 (15 mg kg^-1^) for 15 days reduced MAP without altering BRS. The blockade of muscarinic receptors and nitric oxide synthase by intravenous infusion of atropine and L-NAME, respectively, attenuated cardiovascular effects of LQFM-21. In addition, *ex vivo* experiments showed that LQFM-21 induced an endothelium-dependent relaxation in isolated aortic rings from SHR. This effect was blocked by guanylyl cyclase inhibitor (ODQ) and L-NAME. These findings suggest the involvement of muscarinic receptor and NO/cGMP pathway in the antihypertensive and vasodilator effects of LQFM-21.

## Introduction

Blood pressure control is a complex process that involves variety of organ systems, such as cardiovascular system, central nervous system, adrenal glands, and kidneys (Coffman and Crowley, 2008). Several classes of drugs (beta and alpha blockers, angiotensin converting enzyme inhibitors, calcium channel blockers, diuretics, etc.) are currently being used for the treatment of hypertensive patients in order to reduce cases of morbidity and mortality that are associated with cardiovascular diseases (Law et al., 2009). These classes of drugs are often used in combination for several reasons. However, the cases of side effects that are associated with some of these antihypertensive drugs have led to their use limitations for some patients. Hence, there are needs for the development of new drugs with desirable pharmacological and therapeutic profiles to control arterial hypertension (Manso et al., 2015).

The 5-(1-(3 fluorophenyl)-1H-pyrazol-4-yl)-2H-tetrazole (LQFM-21) is a newly synthesized pyrazole derivative (Ramos Martins et al., 2013). The pyrazole compounds are known to possess hypoglycemic, analgesic, anti-inflammatory, antimicrobial, anticonvulsant, antioxidant, and anti-tumor properties (Gürsoy et al., 2000; Grosse et al., 2014; Liu et al., 2014; Mascia et al., 2014; Küçükgüzel and Şenkardeş, 2015). Moreover, recent studies showed that pyrazole derivatives as neural and inducible nitric oxide synthase (nNOS and iNOS) inhibitors (Carrión et al., 2008), 2,4,5,6-tetrahydrocyclopenta[c]pyrazoles as N-type calcium channel inhibitors (Winters et al., 2014), vasorelaxant and phosphodiesterase inhibitor (Griebenow et al., 2013).

Previous studies have shown that the new LQFM-21 compound has anti-inflammatory, analgesic, and antinociceptive properties (Florentino et al., 2015, 2016, 2017; de Moura et al., 2017). Although this prototype elicited vasorelaxant activity in the isolated arteries (Ramos Martins et al., 2013), the effect of this compound on cardiovascular parameters such as arterial blood pressure, heart rate (HR), and renal and aortic blood flow remains unknown. So, we hypothesized a possible antihypertensive effect of LQFM-21. Therefore, the present study sought to evaluate the antihypertensive and vasodilatory effects of LQFM-21 in spontaneously hypertensive rats (SHR).

## Materials and Methods

### Animals

All experiments were conducted on adult male SHRs or Wistar normotensive rats (NTRs) (280–350 g). Rats were obtained from the central animal house of the Federal University of Goiás. Experimental procedures were designed in strict adherence to the National Health Institute Guidelines for Care and Use of Laboratory Animals and approved by the Ethics Committee on the Use of Animals of Federal University of Goiás (protocol number 034/12).

### Measurement of Systolic Blood Pressure With Tail-Cuff Sphygmomanometer

Spontaneously hypertensive rat(s) and NTR(s) were acclimatized for about 5–6 h in the laboratory at room temperature. Systolic blood pressure (SBP) and HR were measured with a tail-cuff sphygmomanometer with mercury (Harvard Apparatus, MA, United States) in conscious rats pre-warmed at approximately 37°C. This is a non-invasive and indirect method of measuring SBP. The apparatus set up include a restrainer, a tail cuff containing latex tube, and a dual channel recorder. It is pertinent to acknowledge the inherent limitations such as stress that is associated with the use of tail-cuff sphygmomanometer to measure the pressure of conscious SHR and NTR(s).

The vehicle (10% DMSO and 2% Tween in 0.9% NaCl; i.g.) or LQFM-21 (15 mg kg^-1^) was administrated by intragastric catheter (0.3 mL) 2 h prior to the measurements of SBP and HR every 48 h for 15 days. Data were digitized at a frequency of 2000 samples per second using an analog to digital converter (PowerLab 4/25, ML845, ADInstruments, Bella Vista, NSW, Australia).

### Baroreflex Sensitivity Test

The BRS was evaluated through intravenous infusion of phenylephrine (3, 6, and 12 mg kg^-1^; Sigma-Aldrich) and sodium nitroprusside (16, 32, and 64 mg kg^-1^, Sigma-Aldrich) in unanesthetized SHR(s) and NTR(s). BSI index was later calculated as a ratio of changes in HR and MAP.

### Surgical Procedures

Spontaneously hypertensive rat(s) and NTR(s) were anesthetized with halothane (2–3% in O2; Tanohalo; Cristália, Brazil). The right femoral vein and artery were catheterized for drug administration and blood pressure recording, respectively. After catheterization, anesthesia was maintained by intravenous administration of urethane (1.2 g kg^-1^ b.wt., i.v.; Sigma-Aldrich, St. Louis, MO, United States). The trachea was cannulated to facilitate breathing. Subsequently, rats were placed on stereotactic apparatus and retroperitoneal incisions were performed. Miniature ultrasonic transit time flow probes (Transonic Systems Inc., Ithaca, NY, United States) were placed around the left renal artery and abdominal aorta to record renal blood flow (RBF) and aortic blood flow (ABF), respectively. Body temperature was maintained at 37.0 ± 0.5 °C on a thermostatically controlled bench. Following stabilization of cardiovascular parameters, LQFM-21 (0.05; 0.1; 0.2; 0.4 mg kg^-1^) or vehicle (10% DMSO and 2% Tween in 0.9% NaCl) were randomly administered in SHR(s) and NTR(s).

### Recording of Arterial Blood Pressure, Renal and Aortic Blood Flow

The pulsatile arterial pressure (PAP) was continuously recorded through the arterial cannula that was connected to a pressure transducer (MLT0380, ADInstruments, Bella Vista, NSW, Australia) with an amplifier (Bridge Amp, ML221, ADInstruments, Bella Vista, NSW, Australia). The miniatures probes were placed around left renal artery and thoracic aorta and connected to T206 flowmeter (Transonic Systems, Inc., Ithaca, NY, United States) to record the RBF and ABF, respectively. Data were digitized at a frequency of 2000 samples per second using an analogue to digital converter (PowerLab 4/25, ML845, ADInstruments, Bella Vista, NSW, Australia).

### Drugs Administration

#### Effect of L-Name (Nitric Oxide Synthase Inhibitor) and Atropine (Muscarinic Receptors Antagonist) Pretreatment

Spontaneously hypertensive rat(s) group (*n* = 8) were pretreated intravenously with L-Name (0.3 mg kg^-1^, *N_ω_*-nitro-L-arginine methyl ester hydrochloride, Sigma-Aldrich, nitric oxide synthase inhibitor) prior to the measurement of cardiovascular parameters. In a separate experiment, SHR(s) group (*n* = 8) were pretreated intravenously with atropine (0.9 mg kg^-1^, muscarinic receptors antagonist) prior to the measurement of cardiovascular parameters.

### Isolated Aortic Ring Preparation

In other group (*n* = 5), isolated aortic rings of the SHR(s) were used to evaluate the effects of LQFM-21 in rat thoracic aorta. Aortic rings (4 mm) were placed in 9-mL organ bath chambers containing Krebs–Henseleit solution [NaCl (118.06 mmol L^-1^), KCl (4.6 mmol L^-1^), NaHCO_3_ (24.9 mmol L^-1^), MgSO_4_.7H_2_O (2.4 mmol L^-1^), CaCl_2_.2H_2_O (3.3 mmol L^-1^), KH_2_PO_4_ (0.9 mmol L^-1^), and glucose (11.1 mmol L^-1^)] and constant supply of 95% O_2_ and 5% CO_2_ at 37°C. The aortic rings were maintained under a tension of 1.5 g for 1 h to equilibrate. Mechanical activity was recorded isometrically using a data acquisition system (DATAQ Instruments). The effects of LQFM-21 (10^-6^–10^-3^ mol L^-1^) were evaluated in aortic rings [with (E+) or without (E-) endothelium] pre-constricted with phenylephrine (0.1μmol L^-1^). Endothelial integrity was tested with acetylcholine (ACh, 10^-6^ mol L^-1^) in rings previously contracted with Phe (10^-7^mol L^-1^). A relaxation of 80% or more indicated the functional integrity of the endothelium. In order to investigate the action mechanism involved in the effects of LQFM-21, the vessels were pre-treated for 30 min with ODQ (1H-[1,2,4]oxadiazolo[4,3-a]quinoxalin-1-one, Sigma-Aldrich, soluble guanylyl cyclase inhibitor) or L-NAME (nitric oxide synthase inhibitor).

### Data Analysis

Mean arterial pressure (MAP) was calculated with weighted average from PAP signal (2/3 diastolic pressure + 1/3 systolic pressure) using the software PowerLab 4/25 (ML845, ADInstruments, Bella Vista, NSW, Australia). HR was calculated as instantaneous frequency from the PAP signal (PowerLab 4/25, ML845, ADInstruments, Bella Vista, NSW, Australia). Changes in RBF and ABF were calculated as the percentage relative ratio to baseline (% RBF and % ABF). The renal vascular conductance (RVC) and aortic vascular conductance (AVC) were obtained by the ratio of RBF MAP^-1^ and ABF MAP^-1^, respectively. The variations in RVC and AVC were expressed as percentage of change from baseline value (% RVC and % AVC). Statistical analysis was performed using GraphPad Prism version 6.01 (GraphPad Software Inc., San Diego, CA, United States). Data were analyzed by two-way analysis of variance followed by Newman–Keuls *post-test*. The differences in baseline and BSI between groups were analyzed using unpaired Student’s *t*-test. A value of *p* < 0.05 was considered significant.

## Results

### Effects of Chronic Treatment With LQFM-21 on SBP and HR in Unanesthetized SHR and NTR

**FIGURE 1:**
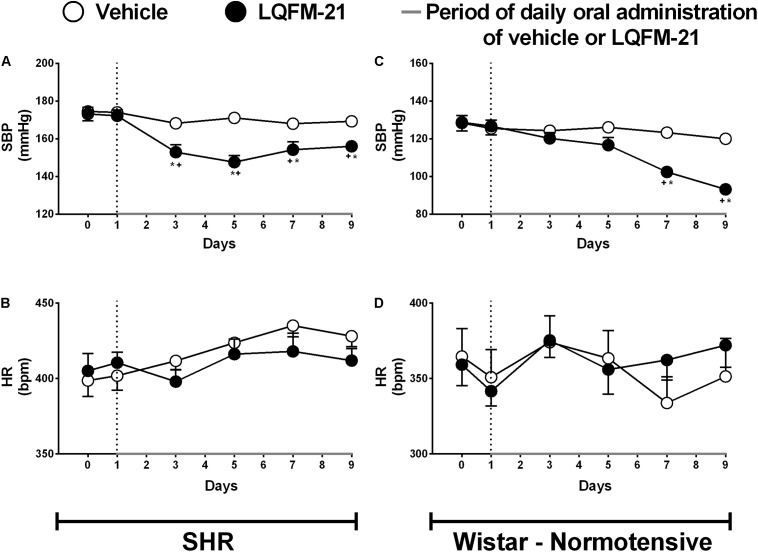
Systolic blood pressure (SBP) in SHR **(A)** and NTR **(C)** unanesthetized animals. Heart rate (HR) in SHR **(B)** and NTR **(D)** unanesthetized animals. Days 0–1 shown values baseline (SBP) while days 1–9 show measurements (every 48 h) SBP values following daily oral administration of the vehicle or LQFM-21 (15 mg⋅kg^-1^). Data are expressed as mean ± SEM (*n* = 10). ^∗^Values significantly different from baseline; ^+^compared to vehicle; *p* < 0.05.

The SHR group (*n* = 10) treated with LQFM-21 (15 mg kg^-1^, i.g.) showed a decrease in SBP, on the 3rd, 5th, 7th, and 9th day of treatment (152.9 ± 3.9; 147.6 ± 3.6; 154.2 ± 4.2; 156.0 ± 2.5 mmHg, respectively, *p* < 0.05; **Figure 1A**) as compared to the baseline (173.2 ± 3.5 mmHg) and to the vehicle (168.2 ± 3.0; 171.1 ± 1.8; 168.0 ± 3.0; 169.3 ± 3.0, respectively; *p* < 0.05; **Figure 1A**). The treatment did not alter HR value (**Figure 1B**).

The NTR(s) (*n* = 10) treated with LQFM-21 (15 mg kg^-1^, i.g.) showed a decrease in SBP, only the 7th and 9th day of treatment (102.4 ± 1.6 and 93.2 ± 2.6 mmHg, respectively, *p* < 0.05; **Figure 1C**) as compared to the baseline (128.7 ± 3.7 mmHg) and to the vehicle (123.3 ± 2.5 and 120.0 ± 1.8, respectively; *p* < 0.05; **Figure 1C**). The treatment did not alter HR value (**Figure 1D**).

**FIGURE 2:**
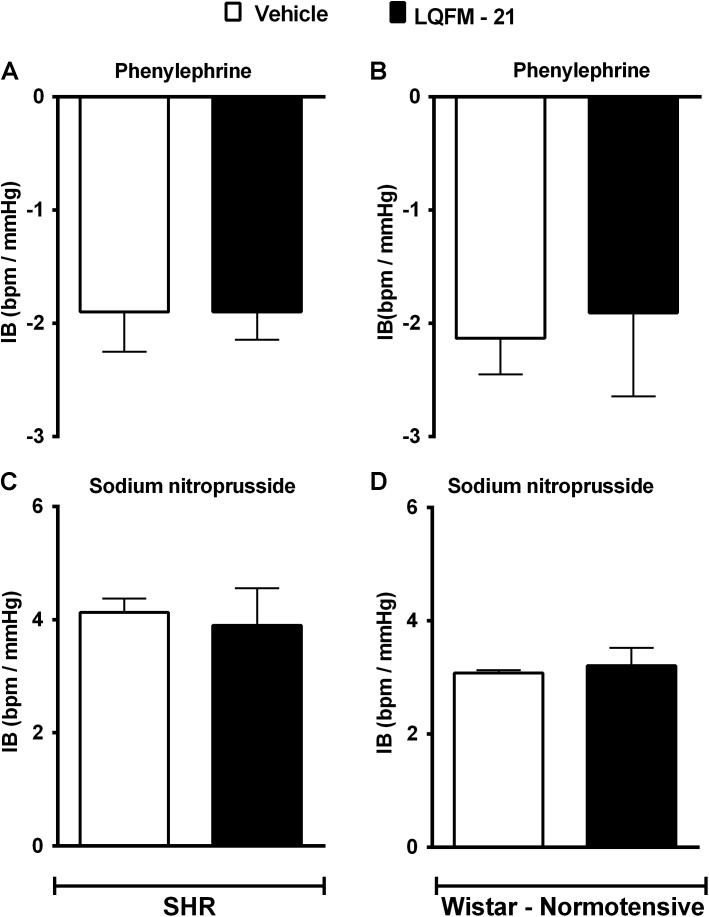
Baroreflex index (BI) of SHR **(A,C)** and NTR **(B,D)** animals induced by phenylephrine and sodium nitroprusside infusions. Vehicle (∙) and LQFM-21 (15 mg⋅kg^-1^) (∙). Data are expressed as mean ± SEM (*n* = 4), with their standard errors represented by vertical bars; *p* < 0.05.

The pre-treatment with LQFM-21 (15 mg kg^-1^, i.g.) did not cause significant changes in the BRS after phenylephrine infusion in SHR(s) (LQFM-21: -1.9 ± 0.2 vs. vehicle: -1.9 ± 0.3 mmHg bpm^-1^; **Figure 2A**; *n* = 4) and NTR(s) (LQFM-21: -1.9 ± 0.4 vs. vehicle: -2.1 ± 0.3 mmHg bpm^-1^; **Figure 2C**; *n* = 4). In addition, the pre-treatment with LQFM-21 (15 mg kg^-1^, i.g.) did not cause significant changes in the BRS after infusion of sodium nitroprusside in SHRs (LQFM-21: -3.9 ± 0.6 vs. -4.1 ± 0.2 bpm mmHg^-1^; **Figure 2B**; *n* = 4) or in NTR(s) (LQFM-21: -3.1 ± 0.3 vs. -3.2 ± 0.0 bpm mmHg^-1^; **Figure 2D**; *n* = 4).

### Effects of Acute Intravenous Infusion of LQFM-21 on MAP, HR, RVC, and AVC in Anesthetized SHR and Normotensive Rats

**FIGURE 3:**
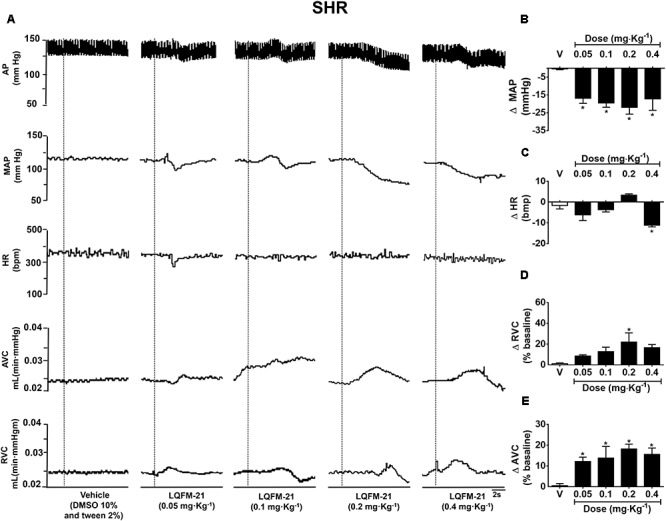
**(A)** Typical tracing of changes in cardiovascular parameters induced by intravenous injection of vehicle or LQFM-21 in anaesthetized SHR. Dashed line indicates the vehicle or LQFM-21 infusion. **(B)** Mean arterial pressure (MAP), **(C)** Heart rate (HR), **(D)** renal vascular conductance (RVC), **(E)** aortic vascular conductance (AVC) in anaesthetized SHR from the response to infusions of vehicle (∙) and LQFM-21 (∙) at doses 0.05, 0.1, 0.2, 0.4 mg⋅kg^-1^. Data are expressed as mean ± SEM, with their standard errors represented by vertical bars (*n* = 6). ^∗^Values significantly different from treated with vehicle; *p* < 0.05.

The typical tracing shows the cardiovascular changes promoted by dose-dependent LQFM-21 administration in SHR (**Figure 3A**). Intravenous administration of LQFM-21 (0.05, 0.1, 0.2, and 0.4 mg kg^-1^) in SHR(s) (*n* = 6) reduced MAP (Δ MAP: -16.8 ± 2.9; -19.5 ± 2.4; -22.0 ± 3.8; -26.6 ± 6.4 mmHg, respectively, *p* < 0.05; **Figure 3B**). LQFM-21 (0.4 mg kg^-1^) reduced HR (ΔHR: -11.2 ± 0.8 bpm, *p* < 0.05; **Figure 3C**). The infusion of LQFM-21 (0.2 mg kg^-1^) increased RVC (Δ% RVC: 22.0 ± 8.8%, *p* < 0.05; **Figure 3D**) and AVC at doses 0.05, 0.1, 0.2, and 0.4 mg kg^-1^ (Δ% AVC: 12.2 ± 2.1; 13.8 ± 5.6; 18.2 ± 2.3; 15.6 ± 3.1, respectively, *p* < 0.05, **Figure 3E**).

**FIGURE 4:**
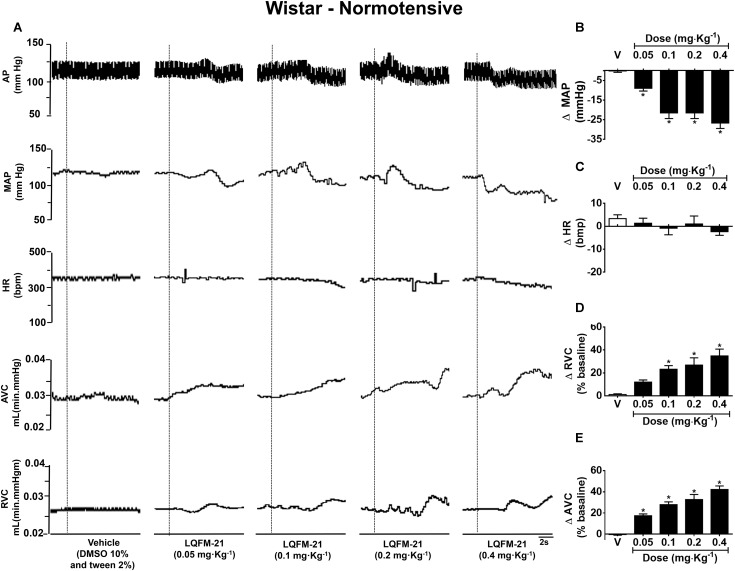
**(A)** Typical tracing of changes in cardiovascular parameters induced by intravenous injection of vehicle or LQFM-21 in anaesthetized NTR. Dashed line indicates the vehicle or LQFM-21 infusion. **(B)** Mean arterial pressure (MAP), **(C)** Heart rate (HR), **(D)** renal vascular conductance (RVC), **(E)** aortic vascular conductance (AVC) in anaesthetized NTR from the response to infusions of vehicle (∙) and LQFM-21 (∙) at doses 0.05, 0.1, 0.2, 0.4 mg⋅kg^-1^. Data are expressed as mean ± SEM, with their standard errors represented by vertical bars (*n* = 6). ^∗^Values significantly different from treated with vehicle, *p* < 0.05.

The typical tracing shows the cardiovascular changes promoted by dose-dependent LQFM-21 administration in NTR(s) (**Figure 4A**). Intravenous administration of LQFM-21 (0.05, 0.1, 0.2, and 0.4 mg kg^-1^) in these rats (*n* = 6) reduced MAP (ΔMAP: -9 ± 1.3; -21.5 ± 2.8; -21.5 ± 2.8; -17.2 ± 2.7 mmHg, respectively, *p* < 0.05; **Figure 4B**). LQFM-21 (0.4 mg kg^-1^) did not change HR (ΔHR: -2.3 ± 1.6 bpm, *p* < 0.05; **Figure 4C**). The infusion of LQFM-21 increased RVC (ΔRVC: 12 ± 1.9; 23.2 ± 3.2; 26.8 ± 6.3; 34.8 ± 5.9 mmHg, respectively, *p* < 0.05; **Figure 4D**) and AVC at doses 0.05, 0.1, 0.2, and 0.4 mg kg^-1^ (Δ% AVC: 17.3 ± 1.7; 27.8 ± 2.6; 32.7 ± 4.8; 42.2 ± 3.4%, respectively, *p* < 0.05, **Figure 4E**).

### Effects of the Nitric Oxide Synthetase Inhibitor on Antihypertensive Response Induced by Acute Infusion of LQFM-21 in Anesthetized SHR

**FIGURE 5:**
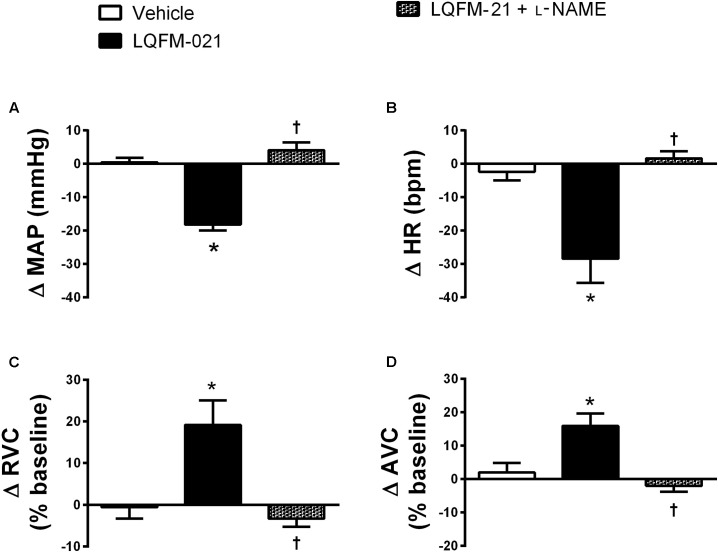
**(A)** Mean arterial pressure (MAP), **(B)** Heart rate (HR), **(C)** renal vascular conductance (RVC), **(D)** aortic vascular conductance (AVC) in anaesthetized rats SHR from the response to infusions of vehicle; LQFM-21 (0.4 mg kg^-1^) and LQFM-21 + L-NAME. Data are expressed as mean ± SEM, with their standard errors represented by vertical bars (*n* = 8). ^∗^Values significantly different from vehicle; ^†^mean values was significantly different from LQFM-21; *p* < 0.05.

The pretreatment with L-NAME attenuated the effect of LQFM-21 on MAP (ΔMAP: -2.4 ± 4.0 mmHg, *p* > 0.05; **Figure 5A**; *n* = 6) and HR (ΔHR: -6.8 ± 8.7 bpm, *p* > 0.05; **Figure 5B**). Renal and aortic vasodilation caused by LQFM-21 was abolished by the pretreatment with L-NAME (Δ% RVC: -2.2 ± 8.2%; Δ% AVC: -5.6 ± 3.8%, *p* > 0.05; **Figures 5C,D**).

### Effects of the Muscarinic Receptors Antagonist on Antihypertensive Response Induced by Acute Infusion of LQFM-21 Anesthetized in SHR

**FIGURE 6:**
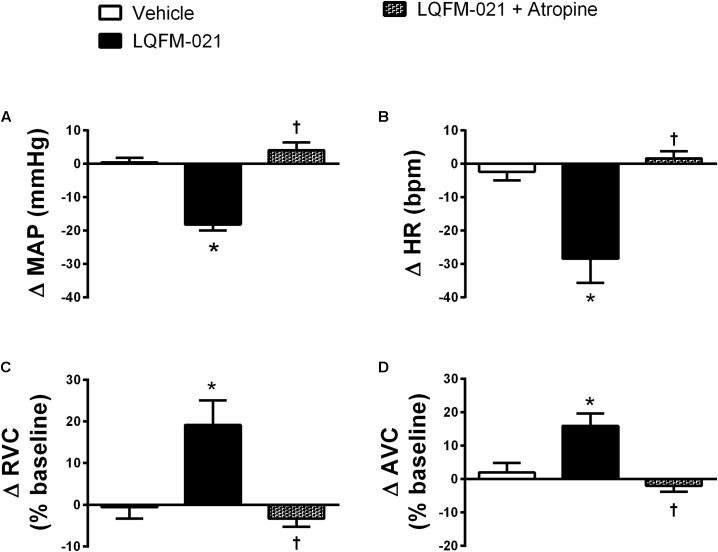
**(A)** Mean arterial pressure (MAP), **(B)** Heart rate (HR), **(C)** renal vascular conductance (RVC), **(D)** aortic vascular conductance (AVC) in anaesthetized rats SHR from the response to infusions of vehicle; LQFM-21 (0.4 mg kg^-1^) and LQFM-21 + atropine. Data are expressed as mean ± SEM, with their standard errors represented by vertical bars (*n* = 8). ^∗^Values significantly different from vehicle; ^†^mean values was significantly different from LQFM-21; *p* < 0.05.

The LQFM-21-induced decrease in MAP and HR was blocked by atropine (ΔMAP: 4.1 ± 2.4 mmHg; ΔHR: 1.6 ± 2.2 bpm, *p* > 0.05; **Figures 6A,B**; *n* = 6). The renal and aortic vasodilation caused by LQFM-21 was inhibited by atropine (Δ% RVC: -3.3 ± 2.0%; Δ% AVC: -2.1 ± 1.8%, *p* > 0.05; **Figures 6C,D**).

### Effect of Guanylyl Cyclase Inhibitor on Vasorelaxation Induced by LQFM-21 in Isolated Aortic Rings of SHR

**FIGURE 7:**
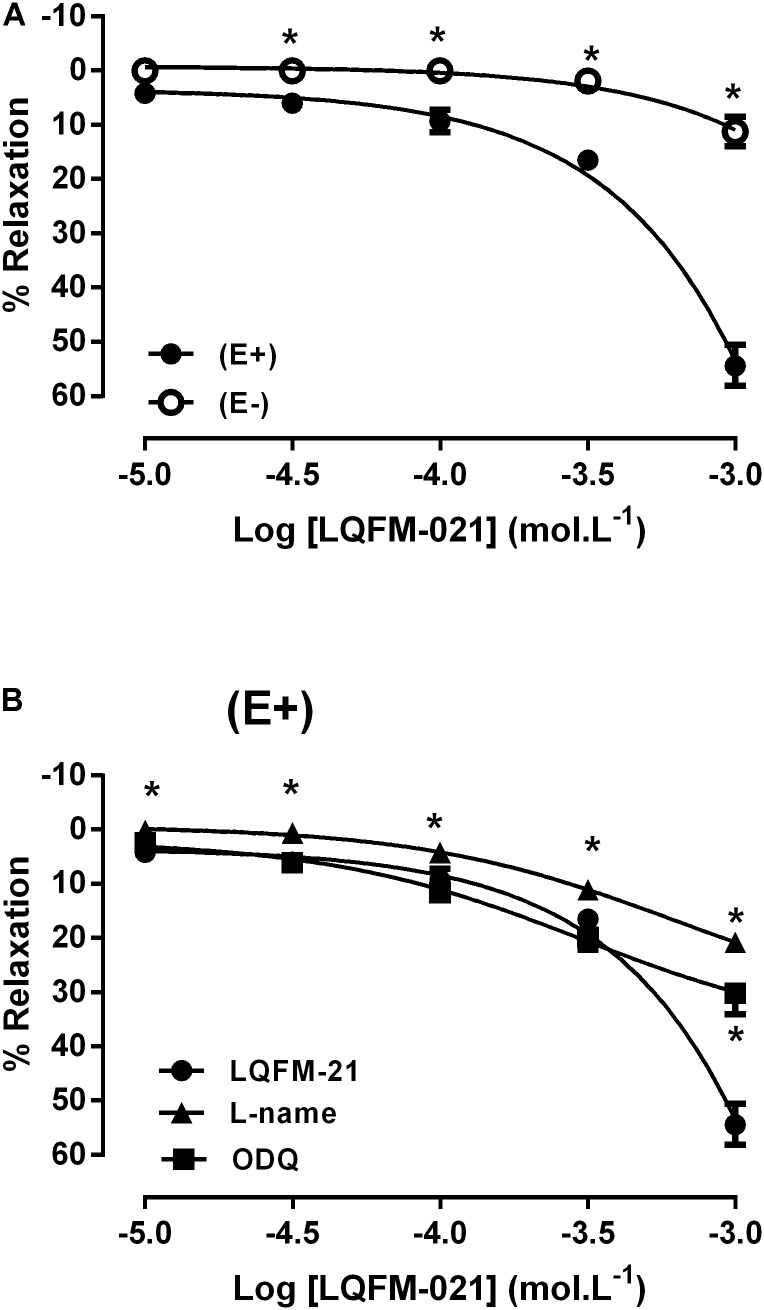
**(A)** Effects of LQFM-21 in aortic rings (E+) or without endothelium (E-), **(B)** effect LQFM-21 on aortic rings of SHR in the absence or presence of L-NAME (10^-6^ mol L^-1^) or ODQ (3.10^-6^ mol L^-1^). Data are expressed as mean ± SEM, (*n* = 5). ^∗^Values significantly different from LQFM-21; *p* < 0.05.

As shown in **Figure 7A**, LQFM-21 induced an endothelium-dependent vascular relaxation (maximal response: 54.4 ± 3.8%, *p* < 0.05; *n* = 5). In endothelium-denuded aorta, LQFM-21 induced only a slight relaxation (maximal response: 11.2 ± 2.7%, *p* > 0.05; **Figure 7A**). The vasorelaxation effect of LQFM-21 was blocked by ODQ (30.2 ± 3.9%, *p* > 0.05; **Figure 7B**) or L-NAME (20.7 ± 0.6%, *p* > 0.05; **Figure 7B**).

## Discussion

The limitations of current antihypertensive therapies have stimulated research and development of new classes of drugs. Some of these new drugs could facilitate better control of blood pressure, high tolerability, and effective prevention of cardiovascular diseases (Ménard, 1993; Tamargo et al., 2015). Synthesis and modification of compounds remain a feasible alternative strategy for new drug development strategies (Wróblewska et al., 2013). Previous data on pyrazole derivatives on voltage-dependent calcium channels (Winters et al., 2014) and vascular function (Carrión et al., 2008; Ramos Martins et al., 2013) suggested potential hemodynamic actions of these class of compounds. However, no study has been conducted in an experimental model of essential hypertension before showed an antihypertensive property of these compounds. So, the present study evaluated the hypotensive property of LQFM-21 and the possible mechanisms of action underlying its hypotensive effects.

To address these aims, the SHR model of hypertension, a gold standard for experimentally studying essential hypertension, was used to investigate the antihypertensive effect of LQFM-21. The spontaneous rise in the blood pressure of SHR shares common characteristics to human hypertension (Head, 1989; Tsuda and Masuyama, 1991). Hypertension in SHR has been attributed to an increase in sympathetic adrenergic activity (Head, 1989; Behuliak et al., 2011). Arribas et al. (1994) demonstrated that β-adrenoceptor-mediated relaxation of the aortic rings in SHR pre-treated with norepinephrine was less than that of NTRs. This difference was associated with impaired endothelial function.

In the present study, intravenous administration of LQFM-21 caused a decrease in MAP, and an increase in vascular conductance in hypertensive and normotensive animals. In addition, chronic oral treatment this compound decreased SBP significantly as compared to vehicles treated groups. Moreover, in SHR models, the blockade of cardiovascular effects of this compound produced by L-NAME and atropine suggest the involvement of nitric oxide synthase and muscarinic receptors in its antihypertensive mechanisms. Furthermore, ACh/NO/cGMP pathway could be proposed as the underlining mechanism for the hypotensive and antihypertensive effects elicited by LQFM-21. This is in agreement with França-Silva et al. (2012b) where authors demonstrated that the NDBP, a new organic nitrate, also reduces blood pressure *via* activation of Ach receptors in both the heart and the vessels.

Atropine is a non-selective antagonist of muscarinic receptors with wide applications particularly as a research tool. In the present study, atropine at the dose of 0.15 mg/kg attenuated the effect of LQFM-21 and thereby suggest the involvement of muscarinic receptors. Subtypes of muscarinic receptors may be linked to excitatory G proteins (M1, M3, and M5), as well as to inhibitory G proteins (M2 and M4). Parasympathetic stimulation and circulating substances often activate muscarinic receptors to elicit reduction of cardiac frequency, force of cardiac contraction, and relaxation of peripheral blood vessels (Ventura et al., 2010). The reduction in these parameters have been associated with the mechanism of antihypertensive drugs. Both cardiac and vascular functions of drugs are important to antihypertensive effects (van Zwieten and Doods, 1995).

The circulating substances and M3 receptors (with greater expression in blood vessels) interaction could activate Gq which in turn induces the activation of phospholipase C. These events promote the hydrolysis of membrane phosphoinositides to diacylglycerol and inositol triphosphate (Sandiford and Slepak, 2009) to induce vasodilation. On the other hand, the activation of inhibitory G protein-bound muscarinic receptor such as M2 (M2 receptor with higher expression in the heart) (Caulfield, 1993) inactivate adenyl cyclase and reduce intracellular levels of cyclic AMP. In addition, studies showed an action of M2 receptors with the activation of endothelium nitric oxide synthase (eNOS) in cardiomyocytes of several mammalian species (Feron et al., 1999; Rocha et al., 2015). Altogether, the signaling pathways involving muscarinic receptors remain important pharmacological targets in the treatment of hypertension.

Currently, over two-thirds of hypertensive cases cannot be treated on just one drug and requires a combination of antihypertensive agents selected from different drug classes (Materson et al., 1993; Hansson et al., 1998; Cushman et al., 2002; Dahlöf et al., 2002; Black et al., 2003). The comparison of “newer” classes of agents, including calcium channel blockers (amlodipine, felodipine, isradipine, nicardipine, nifedipine, and nisoldipine), angiotensin converting enzyme inhibitors (benazepril, captopril, enalapril, fosinopril, lisinopril, moexipril, perindopril, quinapril, ramipril, and trandolapril), alpha-1 receptor blocker (doxazosin, prazosin, and terazosin), and aldosterone receptor blockers (eplerenone and spironolactone), with the “older” diuretics (chlorothiazide, chlorthalidone, hydrochlorothiazide, polythiazide, indapamide, metolazone, and metolazone), and/or beta blockers (atenolol, betaxolol, bisoprolol, metoprolol, nadolol, propranolol, and timolol) (Hansson et al., 1998, 1999, 2000; Brown, 2001; Dahlöf et al., 2002; The Allhat Officers and Coordinators for the Allhat Collaborative Research Group, 2002; Black et al., 2003; Wing et al., 2003) showed that newer classes were neither superior nor inferior to the older ones.

Hence, our findings, which implicate the involvement of nitric oxide synthase and muscarinic receptors in MAP reduction and vasodilatation effect of LQFM-21, provide potential therapeutic windows for the treatment of some hypertensive patients as a monotherapy or in combination with other classes of antihypertensive drugs. This could be of clinical interest in terms of the dose requirement and faster therapeutic effect with little or no side effects. Rapid therapeutic effects of LQFM-21 could greatly improve patient adherence to prescription.

In addition to the mechanisms involved in the vascular function, some antihypertensive drugs could interfere with normal baroreceptors function (Toney et al., 2010) to regulate blood pressure. The reflex response induced by baroreceptor modulates sympathetic activity to the heart, blood vessels, and adrenal medulla. Stimulation of baroreceptors often results in a decrease in sympathetic tone to heart and blood vessels (Cravo et al., 2006). In the present study, the oral chronic administration of LQFM-21 did not alter baroreceptor reflex sensitivity in SHR. This result rule out the involvement of baroreflex pathways.

Moreover, LQFM-21 induced vascular relaxation in isolated aortic rings of SHR. These data suggest a direct effect of this compound on blood vessel. Of note, the vasorelaxation induced by LQFM-21 was blocked by nitric oxide synthase and guanylate cyclase inhibitors. This result is consistent with current *in vivo* blockade of cardiovascular effect by L-NAME. Hence, the vascular effect of this compound could be attributed to NO/cGMP mechanism. Other studies seeking for new compounds have also documented that the NO/cGMP pathway is involved in the hypotensive effect of those compounds (França-Silva et al., 2012a; Dantas et al., 2014; Mendes-Júnior et al., 2015).

Taken together, our results present the first *in vivo* and *ex vivo* antihypertensive and vasorelaxation effects of LQFM-21. These findings suggest the involvement of nitric oxide and muscarinic receptors in the antihypertensive effects of this compound. However, further studies are required to confirm its therapeutic efficacy and safety profile of LQFM-21.

## Author Contributions

NT, PL, LN, JF, PA, NA, and GP conceived and designed the experiments. NT, PL, LN, JF, PA, and NA performed the experiments. NT, PL, LN, JF, PA, NA, LL, AR, CC, VB, RM, and GP analyzed the data and wrote the paper. LL, AR, CC, VB, RM, and GP contributed reagents, materials, and analysis tools.

## Conflict of Interest Statement

The authors declare that the research was conducted in the absence of any commercial or financial relationships that could be construed as a potential conflict of interest.

## References

[B1] Arribas S. Marín J. Ponte A. Balfagón G. Salaices M. (1994). Norepinephrine-induced relaxations in rat aorta mediated by endothelial beta adrenoceptors. Impairment by ageing and hypertension . *J. Pharmacol. Exp. Ther.* 270 520–527. 8071845

[B2] Behuliak M. Pintérová M. Kuneš J. Zicha J. (2011). Vasodilator efficiency of endogenous prostanoids, Ca2+-activated K+ channels and nitric oxide in rats with spontaneous, salt-dependent or NO-deficient hypertension. *Hypertens. Res.* 34 968–975. 10.1038/hr.2011.82 21818109

[B3] Black H. R. Elliott W. J. Grandits G. Grambsch P. Lucente T. White W. B. (2003). Principal results of the controlled onset verapamil investigation of cardiovascular end points (CONVINCE) trial. *JAMA* 289 2073–2082. 10.1001/jama.289.16.2073 12709465

[B4] Brown M. (2001). Principal results from the international nifedipine GITS study: intervention as a goal in hypertension treatment (INSIGHT). *Eur. Heart J. Suppl.* 3 B20–B26. 10.1016/S1520-765X(01)90053-7

[B5] Carrión M. D. López Cara L. C. Camacho M. E. Tapias V. Escames G. Acuña-Castroviejo D. (2008). Pyrazoles and pyrazolines as neural and inducible nitric oxide synthase (nNOS and iNOS) potential inhibitors (III). *Eur. J. Med. Chem.* 43 2579–2591. 10.1016/j.ejmech.2008.01.014 18325637

[B6] Caulfield M. P. (1993). Muscarinic receptors—characterization, coupling and function. *Pharmacol. Ther.* 58 319–379. 10.1016/0163-7258(93)90027-B 7504306

[B7] Coffman T. M. Crowley S. D. (2008). Kidney in hypertension: guyton redux. *Hypertension* 51 811–816. 10.1161/HYPERTENSIONAHA.105.063636 18332286

[B8] Cravo S. L. Rosa D. A. Kalassa F. Korim W. S. Hinrichs J. M. Ferreira-Neto M. L. (2006). Os núcleos vasomotores do bulbo e a regulação cardiovascular: novas evidências e novas questões. *Medicina* 39:89 10.11606/issn.2176-7262.v39i1p89-100

[B9] Cushman W. C. Ford C. E. Cutler J. A. Margolis K. L. Davis B. R. Grimm R. H. (2002). Original papers. *J. Clin. Hypertens.* 4 393–404. 10.1111/j.1524-6175.2002.02045.x 12461301

[B10] Dahlöf B. Devereux R. B. Kjeldsen S. E. Julius S. Beevers G. de Faire U. (2002). Cardiovascular morbidity and mortality in the losartan intervention for endpoint reduction in hypertension study (LIFE): a randomised trial against atenolol. *Lancet* 359 995–1003. 10.1016/S0140-6736(02)08089-3 11937178

[B11] Dantas B. P. Ribeiro T. P. Assis V. L. Furtado F. F. Assis K. S. Alves J. S. (2014). Vasorelaxation induced by a new naphthoquinone-oxime is mediated by NO-sGC-cGMP pathway. *Molecules* 19 9773–9785. 10.3390/molecules19079773 25006785 PMC6270866

[B12] de Moura S. S. de Ávila R. I. Brito L. B. de Oliveira R. de Oliveira G. A. R. Pazini F. (2017). In vitro genotoxicity and in vivo subchronic evaluation of the anti-inflammatory pyrazole compound LQFM021. *Chem. Biol. Interact.* 277 185–194. 10.1016/j.cbi.2017.09.004 28890382

[B13] Feron O. Zhao Y. Y. Kelly R. A. (1999). The ins and outs of caveolar signaling. m2 muscarinic cholinergic receptors and eNOS activation versus neuregulin and ErbB4 signaling in cardiac myocytes. *Ann. N. Y. Acad. Sci.* 874 11–19. 10.1111/j.1749-6632.1999.tb09220.x 10415516

[B14] Florentino I. F. da Silva D. P. Martins J. L. R. da Silva T. S. Santos F. C. Tonussi C. R. (2016). Pharmacological and toxicological evaluations of the new pyrazole compound (LQFM-021) as potential analgesic and anti-inflammatory agents. *Inflammopharmacology* 24 265–275. 10.1007/s10787-016-0282-3 27671330

[B15] Florentino I. F. Galdino P. M. De Oliveira L. P. Silva D. P. B. Pazini F. Vanderlinde F. A. (2015). Involvement of the NO/cGMP/KATP pathway in the antinociceptive effect of the new pyrazole 5-(1-(3-fluorophenyl)-1H-pyrazol-4-yl)-2H-tetrazole (LQFM-021). *Nitric Oxide* 47 17–24. 10.1016/j.niox.2015.02.146 25754796

[B16] Florentino I. F. Silva D. P. Silva D. M. Cardoso C. S. Moreira A. L. E. Borges C. L. (2017). Potential anti-inflammatory effect of LQFM-021 in carrageenan-induced inflammation: the role of nitric oxide. *Nitric Oxide* 69 35–44. 10.1016/j.niox.2017.04.006 28412475

[B17] França-Silva M. S. Luciano M. N. Ribeiro T. P. Silva J. S. F. Santos A. F. França K. C. (2012a). The 2-nitrate-1,3-dibuthoxypropan, a new nitric oxide donor, induces vasorelaxation in mesenteric arteries of the rat. *Eur. J. Pharmacol.* 690 170–175. 10.1016/j.ejphar.2012.06.043 22796675

[B18] França-Silva M. S. Monteiro M. M. O. Queiroz T. M. Santos A. F. Athayde-Filho P. F. Braga V. A. (2012b). The new nitric oxide donor 2-nitrate-1,3-dibuthoxypropan alters autonomic function in spontaneously hypertensive rats. *Auton. Neurosci.* 171 28–35. 10.1016/j.autneu.2012.10.002 23141524

[B19] Griebenow N. Schirok H. Mittendorf J. Straub A. Follmann M. Stasch J.-P. (2013). Identification of acidic heterocycle-substituted 1H-pyrazolo[3,4-b]pyridines as soluble guanylate cyclase stimulators. *Bioorg. Med. Chem. Lett.* 23 1197–1200. 10.1016/j.bmcl.2013.01.028 23385209

[B20] Grosse S. Mathieu V. Pillard C. Massip S. Marchivie M. Jarry C. (2014). New imidazo[1,2-b]pyrazoles as anticancer agents: synthesis, biological evaluation and structure activity relationship analysis. *Eur. J. Med. Chem.* 84 718–730. 10.1016/j.ejmech.2014.07.057 25064349

[B21] Gürsoy A. Demirayak Ş. Çapan G. Erol K. Vural K. (2000). Synthesis and preliminary evaluation of new 5-pyrazolinone derivatives as analgesic agents. *Eur. J. Med. Chem.* 35 359–364. 10.1016/S0223-5234(00)00117-3 10785562

[B22] Hansson L. Hedner T. Lund-Johansen P. Kjeldsen S. E. Lindholm L. H. Syvertsen J. O. (2000). Randomised trial of effects of calcium antagonists compared with diuretics and beta-blockers on cardiovascular morbidity and mortality in hypertension: the Nordic Diltiazem (NORDIL) study. *Lancet* 359 359–365. 10.1016/S0140-6736(00)02526-5 10972367

[B23] Hansson L. Lindholm L. H. Ekbom T. Dahlof B. Lanke J. Schersten B. (1999). Randomised trial of old and new antihypertensive drugs in elderly patients: cardiovascular mortality and morbidity the swedish trial in old patients with Hypertension-2 study. *Lancet* 354 1751–1756. 10.1016/S0140-6736(99)10327-1 10577635

[B24] Hansson L. Zanchetti A. Carruthers S. G. Dahlöf B. Elmfeldt D. Julius S. (1998). Effects of intensive blood-pressure lowering and low-dose aspirin in patients with hypertension: principal results of the hypertension optimal treatment (HOT) randomised trial. *Lancet* 351 1755–1762. 10.1016/S0140-6736(98)04311-6 9635947

[B25] Head R. J. (1989). Hypernoradrenergic innervation: its relationship to functional and hyperplastic changes in the vasculature of the spontaneously hypertensive rat. *Blood Vessels* 26 1–20. 2540863

[B26] Küçükgüzel ŞG. Şenkardeş S. (2015). Recent advances in bioactive pyrazoles. *Eur. J. Med. Chem.* 97 786–815. 10.1016/j.ejmech.2014.11.059 25555743

[B27] Law M. R. Morris J. K. Wald N. J. (2009). Use of blood pressure lowering drugs in the prevention of cardiovascular disease: meta-analysis of 147 randomised trials in the context of expectations from prospective epidemiological studies. *BMJ* 338:b1665. 10.1136/bmj.b1665 19454737 PMC2684577

[B28] Liu Y. Li Y. Chen N. Lv K. Zhou C. Xiong X. (2014). Synthesis and fungicidal activity of novel chloro-containing 1-Aryl-3-oxypyrazoles with an oximino ester or oximino amide moiety. *Molecules* 19 8140–8150. 10.3390/molecules19068140 24941339 PMC6271209

[B29] Manso M. E. G. Biffi E. C. A. Gerardi T. J. (2015). Prescrição inadequada de medicamentos a idosos portadores de doenças crônicas em um plano de saúde no município de São Paulo, Brasil. *Rev. Bras. Geriatr. Gerontol.* 18 151–164. 10.1590/1809-9823.2015.14056

[B30] Mascia M. P. Ledda G. Orrù A. Marongiu A. Loriga G. Maciocco E. (2014). Differential modulation of GABAA receptor function by aryl pyrazoles. *Eur. J. Pharmacol.* 733 1–6. 10.1016/j.ejphar.2014.03.039 24704372

[B31] Materson B. J. Reda D. J. Cushman W. C. Massie B. M. Freis E. D. Kochar M. S. (1993). Single-drug therapy for hypertension in men – A comparison of six antihypertensive agents with placebo. *N. Engl. J. Med.* 328 914–921. 10.1056/NEJM199304013281303 8446138

[B32] Ménard J. (1993). Critical assessment of international clinical development programmes for new antihypertensive drugs. *J. Hypertens. Suppl.* 11 S39–S46. 10.1097/00004872-199312050-00007 8158432

[B33] Mendes-Júnior L. das G. Guimarães D. D. Gadelha D. D. A. Diniz T. F. Brandão M. C. R. (2015). The new nitric oxide donor cyclohexane nitrate induces vasorelaxation, hypotension, and antihypertensive effects via NO/cGMP/PKG pathway. *Front. Physiol.* 6:243. 10.3389/fphys.2015.00243 26379557 PMC4553900

[B34] Ramos Martins D. Pazini F. de Medeiros Alves V. Santana de Moura S. Morais Lião L. Torquato Quezado de Magalhães M. (2013). Synthesis, docking studies, pharmacological activity and toxicity of a novel pyrazole derivative (LQFM 021)–possible effects on phosphodiesterase. *Chem. Pharm. Bull.* 61 524–531. 10.1248/cpb.c12-01016 23649195

[B35] Rocha B. A. M. Barroso-Neto I. L. Teixeira C. S. Santiago M. Q. Pires A. F. Souza L. A. G. (2015). CRLI induces vascular smooth muscle relaxation and suggests a dual mechanism of eNOS activation by legume lectins via muscarinic receptors and shear stress. *Arch. Biochem. Biophys.* 565 32–39. 10.1016/j.abb.2014.11.003 25444858

[B36] Sandiford S. L. Slepak V. Z. (2009). The Gβ 5 -RGS7 complex selectively inhibits muscarinic M3 receptor signaling via the interaction between the third intracellular loop of the receptor and the DEP Domain of RGS7 †. *Biochemistry* 48 2282–2289. 10.1021/bi801989c 19182865 PMC2766429

[B37] Tamargo J. Duarte J. Ruilope L. (2015). New antihypertensive drugs under development. *Curr. Med. Chem.* 22 305–342. 10.2174/0929867321666141106113018 25386825

[B38] The Allhat Officers and Coordinators for the Allhat Collaborative Research Group (2002). Major outcomes in high-risk hypertensive patients randomized to angiotensin-converting enzyme inhibitor or calcium channel blocker vs diuretic: the antihypertensive and lipid-lowering treatment to prevent heart attack trial (ALLHAT). *J. Am. Med. Assoc.* 288 2981–2997. 10.1001/jama.288.23.2981 12479763

[B39] Toney G. M. Pedrino G. R. Fink G. D. Osborn J. W. (2010). Does enhanced respiratory-sympathetic coupling contribute to peripheral neural mechanisms of angiotensin II-salt hypertension? *Exp. Physiol.* 95 587–594. 10.1113/expphysiol.2009.047399 20228120 PMC2978666

[B40] Tsuda K. Masuyama Y. (1991). Presynaptic regulation of neurotransmitter release in hypertension. *Clin. Exp. Pharmacol. Physiol.* 18 455–467. 10.1111/j.1440-1681.1991.tb01478.x 1680586

[B41] van Zwieten P. A. Doods H. N. (1995). Muscarinic receptors and drugs in cardiovascular medicine. *Cardiovasc. Drugs Ther.* 9 159–167. 10.1007/BF00877757 7786837

[B42] Ventura A. L. Abreu P. A. Freitas R. C. Sathler P. C. Loureiro N. Castro H. C. (2010). Colinergic system: revisiting receptors, regulation and the relationship with Alzheimer disease, schizophrenia, epilepsy and smoking. *Arch. Clin. Psychiatry* 37 66–72. 10.1590/S0101-60832010000200007

[B43] Wing L. M. Reid C. M. Ryan P. Beilin L. J. Brown M. A. Jennings G. L. (2003). A comparison of outcomes with angiotensin-converting–enzyme inhibitors and diuretics for hypertension in the elderly. *N. Engl. J. Med.* 348 583–592. 10.1056/NEJMoa021716 12584366

[B44] Winters M. P. Subasinghe N. Wall M. Beck E. Brandt M. R. Finley M. F. (2014). Discovery and SAR of a novel series of 2,4,5,6-tetrahydrocyclopenta[c]pyrazoles as N-type calcium channel inhibitors. *Bioorg. Med. Chem. Lett.* 24 2057–2061. 10.1016/j.bmcl.2014.03.063 24726803

[B45] Wróblewska M. Kasprzyk J. Sączewski F. Kornicka A. Boblewski K. Lehmann A. (2013). Marsanidine and 7-Me-marsanidine, the new hypotensive imidazolines augment sodium and urine excretion in rats. *Pharmacol. Rep.* 65 1025–1032. 10.1016/S1734-1140(13)71085-5 24145098

